# TRPA1 Channels in *Drosophila* and Honey Bee Ectoparasitic Mites Share Heat Sensitivity and Temperature-Related Physiological Functions

**DOI:** 10.3389/fphys.2016.00447

**Published:** 2016-10-05

**Authors:** Guangda Peng, Makiko Kashio, Tianbang Li, Xiaofeng Dong, Makoto Tominaga, Tatsuhiko Kadowaki

**Affiliations:** ^1^Department of Biological Sciences, Xi'an Jiaotong-Liverpool UniversitySuzhou, China; ^2^Division of Cell Signaling, Okazaki Institute for Integrative Bioscience, National Institutes of Natural SciencesOkazaki, Japan; ^3^Department of Physiological Sciences, The Graduate University for Advanced Studies (SOKENDAI)Okazaki, Japan

**Keywords:** TRPA1, heat activation, arthropod, fruit fly, honey bee ectoparasitic mite

## Abstract

The transient receptor potential cation channel, subfamily A, member 1 (TRPA1) is conserved between many arthropods, and in some has been shown to function as a chemosensor for noxious compounds. Activation of arthropod TRPA1 channels by temperature fluctuations has been tested in only a few insect species, and all of them were shown to be activated by heat. The recent identification of chemosensitive TRPA1 channels from two honey bee ectoparasitic mite species (VdTRPA1 and TmTRPA1) have provided an opportunity to study the temperature-dependent activation and the temperature-associated physiological functions of TRPA1 channels in non-insect arthropods. We found that both mite TRPA1 channels are heat sensitive and capable of rescuing the temperature-related behavioral defects of a *Drosophila melanogaster trpA1* mutant. These results suggest that heat-sensitivity of TRPA1 could be conserved between many arthropods despite its amino acid sequence diversity. Nevertheless, the ankyrin repeats (ARs) 6 and 7 are well-conserved between six heat-sensitive arthropod TRPA1 channels and have critical roles for the heat activation of VdTRPA1.

## Introduction

Transient receptor potential (TRP) channels are permeable to cations and share six common transmembrane segments that form sensor and pore domains. Intriguingly, TRP channels display diverse cation selectivities and activation mechanisms unlike many other cation channels (Venkatachalam and Montell, 2007; Julius, 2013). Thus, TRP channels are considered as the primary signal integrators for various sensory perception such as vision, thermosensation, olfaction, hearing, taste sensation, and mechanosensation (Damann et al., 2008). TRP channels also function for individual cells to detect changes in temperature, osmolarity, and fluid flow in their local environment (Venkatachalam and Montell, 2007; Nilius and Owsianik, 2011). Although significant progress has been made to understand the activation mechanisms and physiological functions of TRP channels in human and model organisms, we still do not fully understand how TRP channels have evolved in diverse animal species. Nevertheless, there is evidence that suggests evolutionary plasticity and dynamics of metazoan TRP channels at multiple levels. This has been shown by gene gain and loss, amino acid substitutions, and transcription and pre-mRNA splicing (Kadowaki, 2015). Thus, in contrast to several conserved cation channels, such as K^+^ channels, TRP channels have undergone more rapid evolution (Kadowaki, 2015; Saito and Tominaga, 2015). TRP channels appear to be under more relaxed conditions since they are only expressed in subsets of neurons and cells.

The metazoan TRP superfamily is classified into eight subfamilies—TRPA, TRPC, TRPM, TRPML, TRPN, TRPP, TRPV, and TRPVL—according to the phylogenetic tree based on the amino acid sequences of six transmembrane segments (Peng et al., 2015b). Among them, TRPA1 specifically contains 14–16 ankyrin repeats (ARs) in the *N*-terminus. ARs are 33 residue motifs consisting of pairs of antiparallel α-helices connected by β-hairpin motifs. Structure of human TRPA1 revealed by cryo-EM is similar to that of TRPV1 by forming a homotetramer to contain the pore with upper and lower gates (Cao et al., 2013; Liao et al., 2013; Paulsen et al., 2015). The physiological functions of TRPA1 have been characterized using genetically tractable model organisms. TRPA1 is activated by nociceptive thermal (either heat or cold) and chemical stimuli, demonstrating that it functions for nociception and inflammatory pain (Nilius et al., 2012). Furthermore, the roles in temperature control of rhythmic activity, promoting longevity at cold temperatures, and induction of embryonic diapause in progeny were reported with *Drosophila melanogaster, Caenorhabtitis elegans*, and *Bombyx mori*, respectively (Xiao et al., 2013; Sato et al., 2014; Das et al., 2016).

The evolution of TRPA1 channels has also been shown to be plastic throughout animal evolution. For example, the genome of the sponge *Amphimedon queenslandica* contains 12 *TRPA1* genes, but they are absent in the water flea *Daphnia pulex* and in hymenopteran insects (bees, wasps, and ants; Peng et al., 2015b). The gene gain and loss of *TRPA1* could have happened within specific animal lineages. Menthol activates human TRPA1 in a concentration-dependent manner, but its action on mouse TRPA1 is bimodal; it activates TRPA1 at low concentrations but inhibits it at high concentrations (Karashima et al., 2007; Xiao et al., 2008). *D. melanogaster* TRPA1 (DmTRPA1) appears to be insensitive to menthol at any concentration (Xiao et al., 2008). Residues S876 and T877 in the transmembrane segment 5 of mouse TRPA1 are critical for activation by menthol, and these are conserved in human but not the Fugu fish, fruit fly, or mosquito TRPA1 (Xiao et al., 2008). Meanwhile, G878 of mouse TRPA1 is critical for channel inhibition at high concentrations, and it is substituted by valine in human TRPA1 (Xiao et al., 2008). Regarding the temperature sensitivity of TRPA1, it has been shown that rodent TRPA1 is cold-activated, whereas primate TRPA1 is insensitive to temperature fluctuations (Chen et al., 2013). Furthermore, it has been recently shown that three single-point mutations in the AR6 of mouse TRPA1 are individually sufficient to make the channel heat-activated without affecting its chemical sensitivity (Jabba et al., 2014). These results demonstrate that minimal changes in the protein sequence of a TRP channel can dramatically change its temperature sensitivity. Above two are the examples of TRPA1 plasticity to modify the channel properties by amino acid substitutions.

TRPA1 has been characterized in a limited number of species. For example, only a few insect TRPA1 channels (fruit fly, malaria mosquito, silk moth, cotton bollworm, and green plant bug) have been characterized among the arthropods. Among those insect TRPA1 channels, DmTRPA1 has been best characterized regarding its channel properties and physiological functions. DmTRPA1 is expressed in chemosensory neurons of the labral sense organ and labellum (Kang et al., 2010, 2012; Kim et al., 2010), as well as in lateral (LC), ventral (VC), and anterior cell (AC) neurons of the brain (Hamada et al., 2008), and in the class IV multidendritic (mdIV) neurons of larvae (Zhong et al., 2012). DmTRPA1 is sensitive to nociceptive compounds such as electrophiles and insect repellent (Kang et al., 2010; Du et al., 2015), as well as to heat (Kang et al., 2012). Intriguingly, its expression and channel gating profiles have been shown to be isoform-specific (Kang et al., 2012; Zhong et al., 2012). DmTRPA1 has multiple roles for temperature-related behaviors, such as temperature control of rhythmic activity (Das et al., 2016), thermal nociception (Neely et al., 2011; Zhong et al., 2012), and thermotactic behaviors (Hamada et al., 2008; Kwon et al., 2008). It was shown that the larval thermal nociception behavior depends on the mdIV neurons (Zhong et al., 2012) and the adult thermotactic behavior depends on the AC neurons of brain (Hamada et al., 2008).

We have recently identified and generally characterized the chemical responses of TRPA1 channels in *Varroa destructor* (VdTRPA1) and *Tropilaelaps mercedesae* (TmTRPA1), two major ectoparasitic mite species of the honey bee. The mite TRPA1 channels share some common chemo-sensitivities (for example, electrophiles) with fruit fly and human TRPA1 channels, but have also acquired specific chemo-sensitivities (for example, α-terpineol) during their evolution. These TRPA1 channels are widely present in body parts of mites; however, some of the isoforms are highly expressed in the forelegs or in the main body (idiosoma; Peng et al., 2015a; Dong et al., 2016). These mite TRPA1 channels gave us an opportunity to study the temperature-dependent activation and temperature-associated physiological functions in non-insect arthropod TRPA1 channels. Although we have briefly reported heat activation of TmTRPA1 (Dong et al., 2016), in this report we characterize the thermo-sensitivity of VdTRPA1 in detail as well as the outcomes of introducing either VdTRPA1 or TmTRPA1 to a *D. melanogaster trpA1* mutant. We aimed to test the conservation of heat activation as well as temperature-related physiological functions between TRPA1 channels in two mite species and *Drosophila*. We then attempted to identify the amino acid sequence that is potentially important for the heat activation of six arthropod TRPA1 channels. Additionally, we discuss the conservation of TRPA1 as the heat sensor among arthropods.

## Materials and methods

### Ca^2+^-imaging method

For Ca^2+^-imaging, 1 μg of VdTRPA1 expression vector and 0.1 μg of pCMV-DsRed-express vector were transfected to HEK293 cells in OPTI-MEM medium (Life Technologies) using Lipofectamine Plus reagents (Life Technologies). After incubating for 3–4 h at 37°C, cells were reseeded on cover glasses and further incubated at 33°C. The cells were used for the experiments at 20–40 h after transfection. Transfected HEK293 cells on a cover glass were incubated in culture media containing 5 μM fura-2 AM (Life Technologies) at 33°C for 1–2 h. The cover glass was washed and fura-2 fluorescence was measured in a standard bath solution containing (in mM) 140 NaCl, 5 KCl, 2 MgCl_2_, 2 CaCl_2_, 10 HEPES, and 10 glucose, pH 7.4 adjusted with NaOH. Calcium chloride was omitted and 5 mM EGTA was added in the calcium-free bath solution. A cover glass was mounted in a chamber (RC-26G, Warner Instruments Inc.) connected to a gravity flow system to deliver hot or cold bath solution. The emitted fluorescence (510 nm) by 340 and 380 nm were measured by CCD camera (CoolSNAP ES, Roper scientific photometrics). The measurement was repeated at least with two cover glasses. Data were acquired and analyzed by IPlab software (Scanalytics Inc.).

### Electrophysiology

For patch-clamp recording, 1 μg VdTRPA1 expression vector and 0.1 μg pGreen Lantern 1 vector were transfected to HEK293 cells cultured on 35-mm dishes using Lipofectamine Plus reagents. The standard bath solution containing 2 mM CaCl_2_ for the whole-cell patch-clamp methods was the same as that used for the Ca^2+^ imaging experiments. The CsAspartate/Ca^2+^(−) pipette solution for the whole-cell patch-clamp methods contained (in mM) 120 Aspartate, 10 CsCl, 1 MgCl_2_, 5 EGTA, and 10 HEPES at pH 7.4, adjusted with CsOH. Whole-cell recording data were sampled at 10 kHz and filtered at 5 kHz for analysis (Axopatch 200B amplifier with pClamp software; Molecular Devices). The membrane potential was clamped at −60 mV in whole-cell configuration. Shift in liquid junction potential during heating (~2–4 mV) was not corrected in the analysis. All of the patch-clamp methods were performed at RT except during the cold and heat stimulation experiments. For heat stimulation, a preheated solution was perfused with gravity, reaching to maximum of 40°C. For cool stimulation, a prechilled solution in ice was used as described for the heat stimulation. The temperature was monitored with a thermocouple (TA-30; Warner Instruments) placed within 100 μm of the patch-clamped cell.

### Genetics

UAS-DmTRPA1, UAS-VdTRPA1L, UAS-VdTRPA1S, and UAS-TmTRPA1b fruit flies were generated by integrating the transgenes to 68A4 by PhiC31 integrase-mediated recombination event as previously reported (Peng et al., 2015a; Dong et al., 2016). These transgenes were driven by *dtrpA1-Gal4* (Rosenzweig et al., 2005; Kim et al., 2010) for the expression in chemosensory neurons of the labral sense organ and labellum and AC, LC, and VC neurons of the brain or *ppk1.9-Gal4* (Ainsley et al., 2008) for the expression in the mdIV neurons of larva under *trpA1*^1^ background (Kwon et al., 2008).

### Assay for thermotactic behavior of *D. melanogaster*

To assay the temperature preference of fruit flies, a temperature gradient of 14–37°C with a slope of 0.94°C/cm was produced in an aluminum block (27 long × 15 wide × 2.5 cm high) as previously reported (Sayeed and Benzer, 1996). Thermometers were embedded in the block every 2.4 cm, and the gradient was established using a cold circulating water chamber and a hot probe at each end. The aluminum block was covered with moist paper to maintain a uniform relative humidity along the gradient. This paper was divided into 20 observation fields with a black pencil for recording the distribution of fruit flies. A glass plate with three separate lanes was placed 5 mm above the block, creating suitable corridors for fruit flies to migrate. Forty to Fifty fruit flies per lane were placed in the middle of testing arena around 25°C between the aluminum block and the glass plate, allowed to migrate for 3 h, and photographed every 10 min with a digital camera. When the positions of fruit flies in the apparatus were stabilized between 1.5 and 2.5 h (This time period did not differ between the experimental groups), the number of fruit flies in each observation field was counted and divided by the total number of fruit flies in the entire area. Prior to introducing fruit flies into the temperature gradient apparatus, they were placed in plastic vials and cooled on ice until they stopped moving. All experiments were performed in a room where the temperature was kept constant at 25°C.

### Assay for thermal nociception behavior of *D. melanogaster* larvae

To test the thermal nociception behavior of *D. melanogaster* larvae, the actively wandering 3rd instar larvae were collected from vials and transferred to 35 mm plastic petri dishes with water to support exhibiting the full rolling response. Larvae were touched laterally in abdominal segments (A4–A6) with the hot probe. In all experiments, 20–40 larvae were tested per group in four replicates. The behavioral responses were recorded and analyzed as described (Tracey et al., 2003; Neely et al., 2011; Zhong et al., 2012). To calculate the median of response latency of each genotype, the latency of larvae responding over 10 s was considered as 10 even though some of the larvae never responded to touching by the hot probe.

### Construction of chimeric VdTRPA1L channels

Three chimeric VdTRPA1L channels swapped with the AR6, AR7, or ARs6 and 7 of HsTRPA1 were constructed by overlap extension PCR (Lee et al., 2004). Four VdTRPA1L DNA fragments containing 9 bp from 5′ to 3′ ends of HsTRPA1 ARs 6 and 7 were PCR amplified and purified. Similarly, three HsTRPA1 DNA fragments containing 9 bp from 5′ to 3′ ends of VdTRPA1L ARs 5, 6, 7, and 8 were PCR amplified and purified. Three of above purified PCR products were mixed and PCR amplified using the outer primers derived from VdTRPA1L sequence. The PCR products and a mammalian expression vector containing VdTRPA1L (Peng et al., 2015a) were digested with Hind III and ligated. The resulting constructs were verified by sequencing.

### Statistics

**Figure 1:**
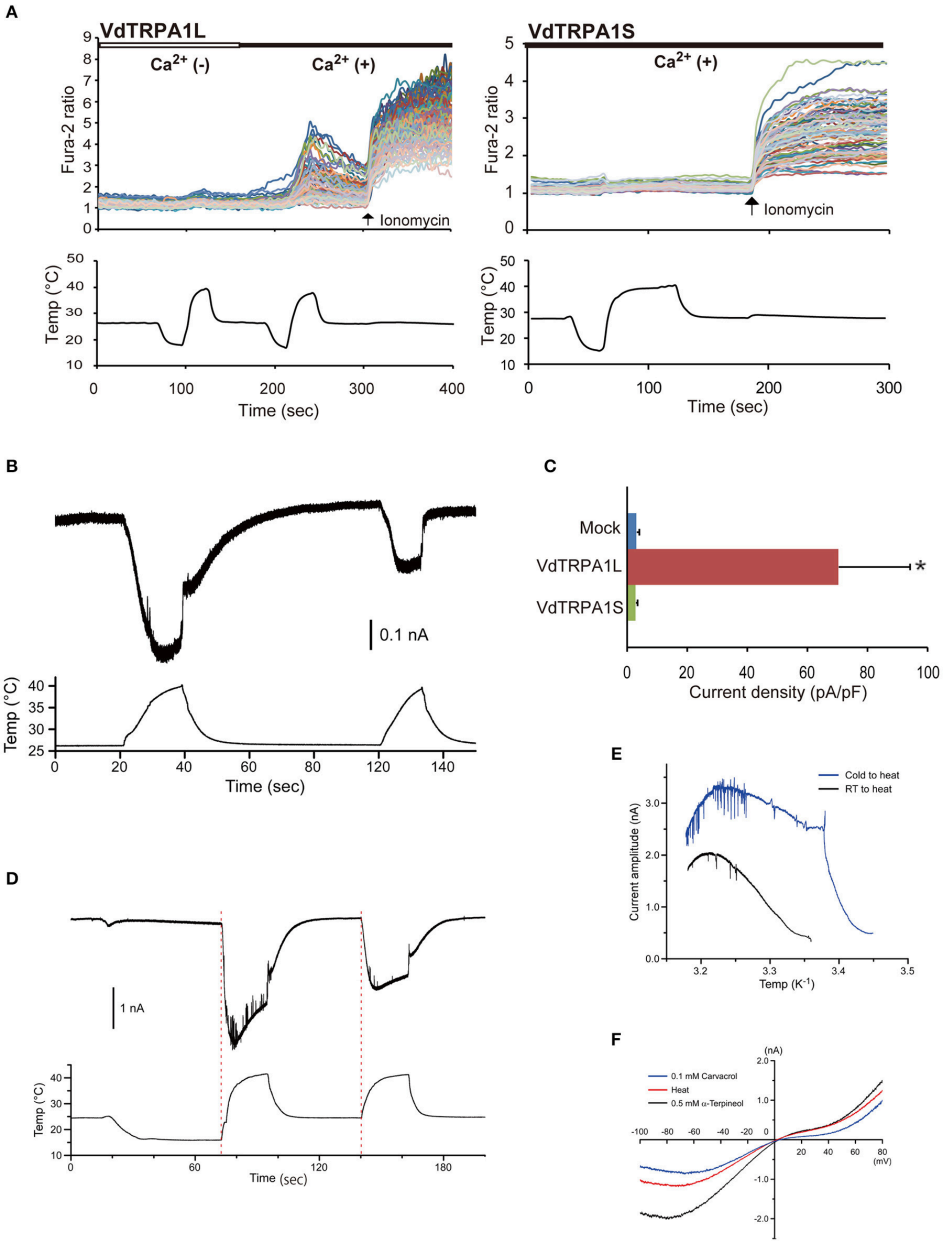
**Heat activation profile of VdTRPA1L. (A)** The upper traces indicate the changes of intracellular calcium level in VdTRPA1L- and VdTRPA1S-expressing cells (84 and 54 DsRed-positive cells on one cover glass, respectively) on temperature fluctuation in the absence [white bar, Ca^2+^ (−)] or presence [black bar, Ca^2+^ (+)] of extracellular calcium. Each line represents the intracellular calcium level in the individual cell measured by calcium imaging. The arrows show the time points when 5 μM ionomycin was added. The lower traces show the changes of bath temperature by time (sec, second). **(B)** Heat activation of VdTRPA1L analyzed by whole-cell patch clamp recording. **(C)** Heat-activated current density of mock-transfected cells, VdTRPA1L-, and VdTRPA1S-expressing cells measured by patch-clamp recording. The mean value with error bar (± SEM) is indicated for each sample (*n* = 6 for VdTRPA1L and *n* = 4 for mock-transfected and VdTRPA1S). *p*-value for VdTRPA1L-expressing cells compared to mock-transfected cells is < 0.04 (^*^). **(D)** The representative trace indicates the activation currents of VdTRPA1L elicited by two different heat applications, 16–42°C (the first stimulation) and 24–42°C (the second stimulation). The red dotted lines show the apparent initiation points of the currents. The upper and lower traces show the changes of inward current and the changes of bath temperature by time. **(E)** Arrhenius plots for the heat-evoked VdTRPA1L currents shown in **(D)**. Blue and black lines represent the current amplitudes generated by heat stimulation from 16°C (Cold to heat) to 24°C (RT to heat), respectively. **(F)** Current–voltage relationship of heat (red line)-, 0.5 mM α-terpineol (black line)-, or 0.1 mM carvacrol (blue line)-evoked current exhibits a positive reversal potential. We corrected the liquid junction potential (ΔJPH) between extracellular and intracellular solution but not the heat-dependent shifts of ΔJPH in the plot. The data of α-terpineol and carvacrol were reproduced from previous report (Peng et al., 2015b).

**Figure 2:**
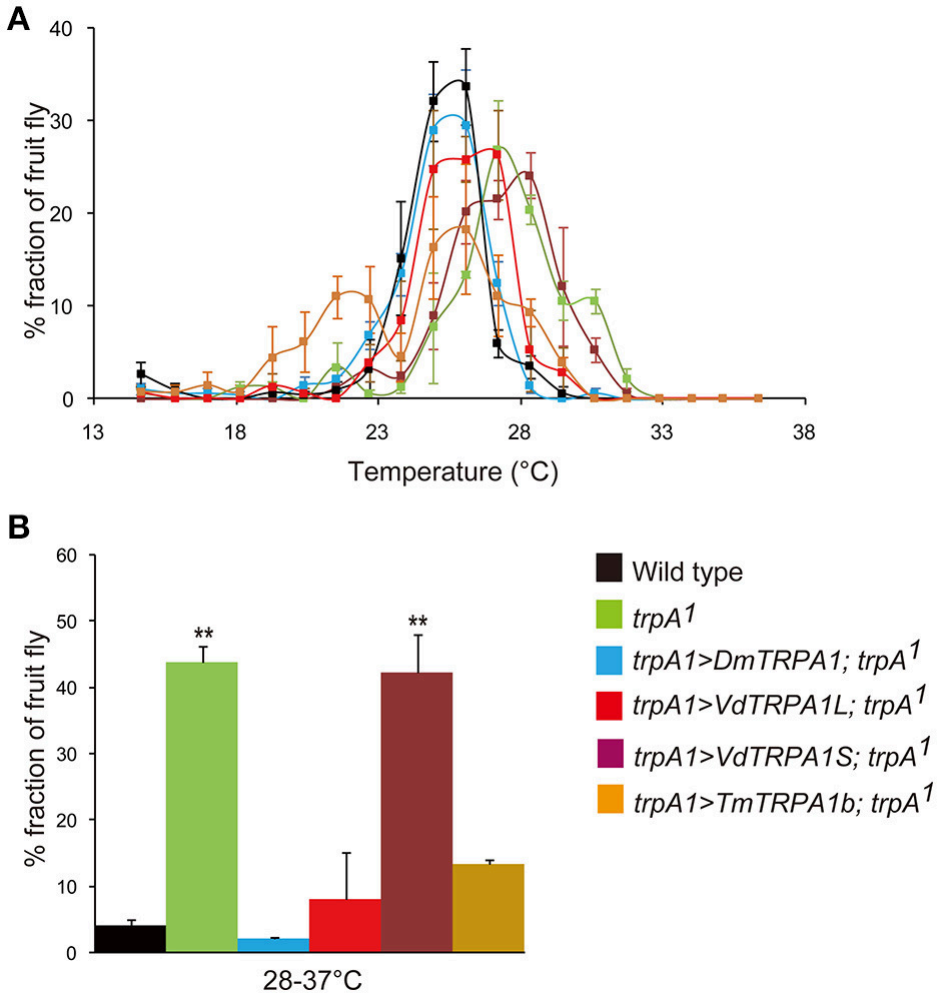
**VdTRPA1L and TmTRPA1b are able to complement the functions of DmTRPA1 for thermotactic behavior of *Drosophila melanogaster*. (A)** The distribution of wild type, trpA1^1^, and trpA1^1^ expressing either DmTRPA1 (trpA1>DmTRPA1; trpA1^1^), VdTRPA1L (trpA1>VdTRPA1L; trpA1^1^), VdTRPA1S (trpA1>VdTRPA1S; trpA1^1^), or TmTRPA1b (trpA1>TmTRPA1b; trpA1^1^) under trpA1-Gal4 was recorded along a thermal gradient (14–37°C). The recording was repeated three times for each group. The mean value with error bar (± SEM) is shown for each temperature section. **(B)** The percentage of fruit flies in the area of 28–37°C (Wild type: 4.1 ± 0.9%; trpA1^1^: 43.8 ± 2.5%; trpA1^1^ expressing DmTRPA1: 2.0 ± 0.4%; trpA1^1^ expressing VdTRPA1L: 8.0 ± 7.1%; trpA1^1^ expressing VdTRPA1S: 42.2 ± 5.8%; trpA1^1^ expressing TmTRPA1b: 13.3 ± 0.8%) of the thermal gradient. Asterisks (^**^) are significantly different from wild type, and *P*-values for both trpA1^1^ and trpA1^1^ expressing VdTRPA1S are < 0.00003 and < 0.00007, respectively.

**Figure 3:**
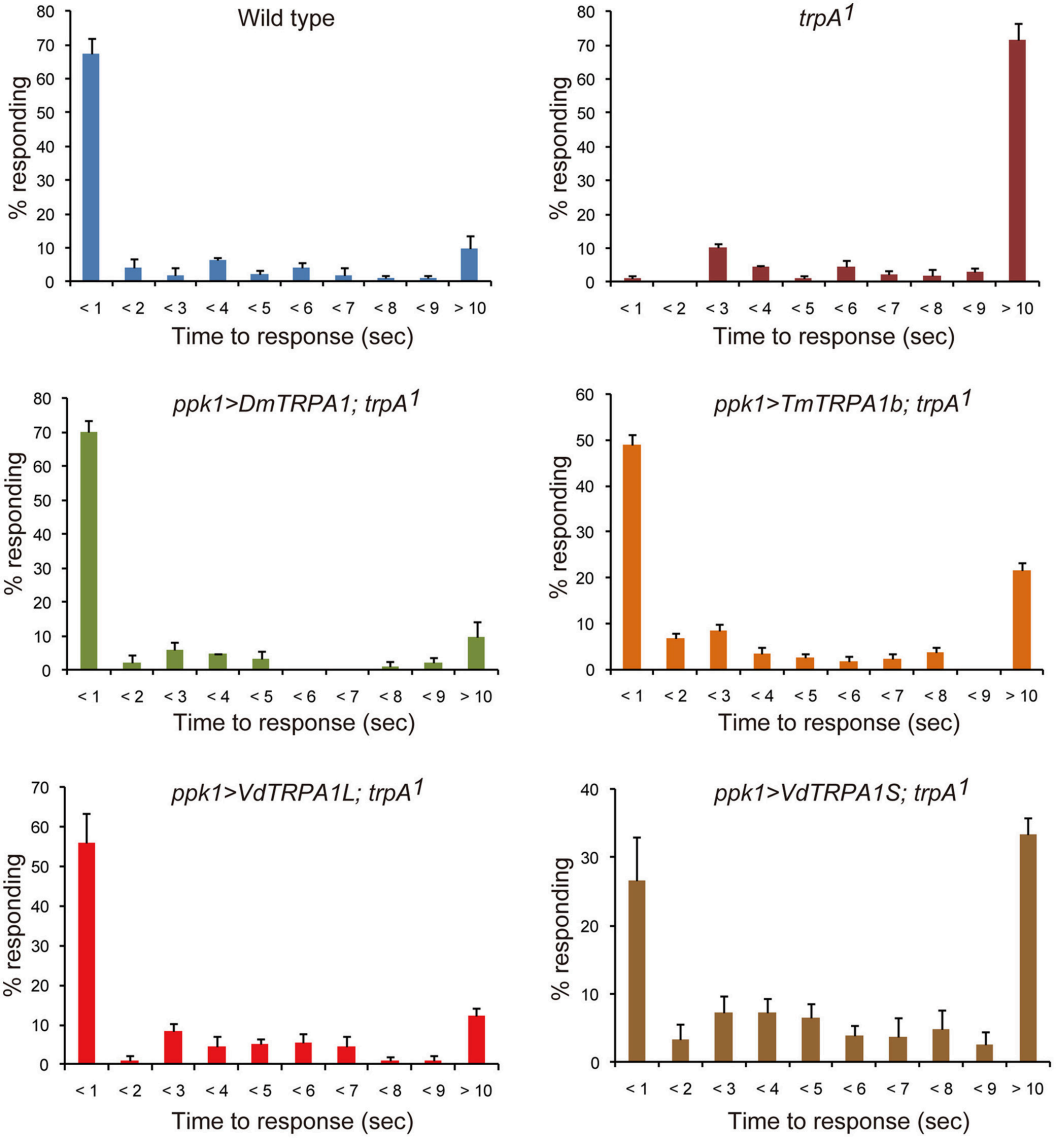
**VdTRPA1L and TmTRPA1b are able to complement the functions of DmTRPA1 for thermal nociception behavior of *Drosophila melanogaster* larvae**. Wild type (*n* = 92), trpA11 (*n* = 90), and trpA1^1^ expressing either DmTRPA1 (ppk1>DmTRPA1; trpA1^1^, *n* = 83), VdTRPA1L (ppk1>VdTRPA1L; trpA1^1^, *n* = 124), VdTRPA1S (ppk1>VdTRPA1S; trpA1^1^, *n* = 139), or TmTRPA1b (ppk1>TmTRPA1b; trpA1^1^, *n* = 162) under ppk1.9-Gal4 were tested for their response to high temperature using 46°C probe. The percentage of responding larvae for each genotype is shown at each second within 9 s and over 10 s (mean value ± SEM). *p*-values for trpA1^1^ and trpA1^1^ expressing VdTRPA1S compared to wild type are < 0.02 and < 0.03, respectively. *p*-values for wild type and trpA1^1^ expressing DmTRPA1 are < 0.02, and *P*-values for trpA1^1^ expressing either VdTRPA1L, VdTRPA1S, or TmTRPA1b are < 0.03 compared to trpA1^1^.

Heat-activated current density measured by patch-clamp recording and thermotactic behavior of fruit flies with different genotypes were analyzed by one-way ANOVA followed by the Dunnett *post-hoc* test (two-tailed) (Figures 1C, 2B). A Kruskal-wallis non-parametric test for median comparison followed by the Steel *post-hoc* test was used for statistical analysis of thermal nociception behavior of fruit fly larvae with different genotypes (Figure 3).

## Results

### Heat activation of VdTRPA1L but not VdTRPA1S

There are two VdTRPA1 isoforms, VdTRPA1L and VdTRPA1S with 15 and 10 ARs, respectively. We previously showed that various plant oil-derived tick repellents activated VdTRPA1L but not VdTRPA1S (Peng et al., 2015a). Calcium imaging technique was used to measure the relative changes of intracellular calcium levels of HEK293 cells expressing either VdTRPA1L or VdTRPA1S by temperature fluctuations. Activation of the channel is expected to increase the intracellular calcium levels by influx of extracellular calcium. As shown in Figure 1A, increased temperature elevated the relative intracellular calcium levels of cells expressing VdTRPA1L but not VdTRPA1S. Furthermore, this effect was observed only in the presence of extracellular calcium, confirming the influx of extracellular calcium was essential. We also verified the VdTRPA1L heat activation using whole-cell patch clamp recording (Figures 1B,C). The activated current of VdTRPA1L reduced by the second heat stimulation relative to the first stimulation, suggesting that the channel is rapidly desensitized (Figure 1B). Low temperature did not increase the relative intracellular calcium levels of cells expressing both channels (Figure 1A). The activated current of VdTRPA1L was immediately detected upon temperature increase regardless of the initial holding temperature either 24°C or 16°C (Figure 1D). Furthermore, the specific transition temperature of channel activation was absent for both initial holding temperatures in the Arrhenius plots (Figure 1E). VdTRPA1L currents activated by either heat, 0.5 mM α-terpineol, or 0.1 mM carvacrol stimulation showed similar non-linear I-V relation (Figure 1F; Peng et al., 2015a). The reversal potentials of heat, 0.5 mM α-terpineol, and 0.1 mM carvacrol were −0.6 ± 2.2, 1.2 ± 0.8, and 3.3 ± 0.7 mV (mean ± SEM, *n* = 4, no statistical differences by ANOVA; Peng et al., 2015a), respectively. These results demonstrate that opened VdTRPA1L channel shows similar ion permeability regardless of stimulus.

### VdTRPA1L and TmTRPA1b, but not VdTRPA1S, effectively rescue the impaired thermotactic and thermal nociception behaviors of a *D. melanogaster* trpA1 mutant

Above results revealed that VdTRPA1L, but not VdTRPA1S, is activated by heat. We, therefore, tested the thermotactic behaviors of wild-type, *trpA1*^1^ (Kwon et al., 2008), and *trpA1*^1^ adults expressing *DmTRPA1, VdTRPA1L, VdTRPA1S*, or *TmTRPA1b* under *trpA1-Gal4* (Rosenzweig et al., 2005; Kim et al., 2010) by using an aluminum block at a temperature gradient of 14–37°C (Sayeed and Benzer, 1996). TmTRPA1b is one of the heat-sensitive TmTRPA1 isoforms, and it is highly expressed in the forelegs of *T. mercedesae*, which are functionally similar to insect antennae (Dong et al., 2016). Although wild-type adult fruit flies preferred temperatures around 25–26°C and avoided temperatures higher than 28°C (Figures 2A,B), *trpA1*^1^ adults preferred high temperatures, and increased numbers of flies were found in the test zones where temperatures were higher than 28°C (Figures 2A,B; Hamada et al., 2008). However, this behavioral defect was rescued by expressing *DmTRPA1, VdTRPA1L*, or *TmTRPA1b*, but not *VdTRPA1S* (Figures 2A,B). *trpA1*^1^ expressing *TmTRPA1b* exhibited two peaks of distribution at around 22–23°C and 25–26°C, and appeared to be broader than those of wild-type and *trpA1*^1^ expressing *DmTRPA1* across the tested temperatures. A slightly broader distribution was also observed with *trpA1*^1^ expressing *VdTRPA1L*.

We also tested the thermal nociception behaviors of the wild-type, *trpA1*^1^, and *trpA1*^1^ larvae expressing *DmTRPA1, VdTRPA1L, VdTRPA1S*, or *TmTRPA1b* under *ppk1.9-Gal4* (Ainsley et al., 2008). For this, we examined the stereotyped nocifensive escape locomotion behavior upon touch with a 46°C probe (Tracey et al., 2003; Neely et al., 2011; Zhong et al., 2012). The above-mentioned *TRPA1* genes were expressed in the mdIV neurons, which function as the polymodal nociceptors of larvae, under the *ppk1.9-Gal4* promoter. As previously reported, most of the wild type larvae initiated the escape behavior within 1 s; however, *trpA1*^1^ larvae displayed a significantly delayed response (Figure 3; Neely et al., 2011; Zhong et al., 2012). Many of the mutants failed to initiate the escape behavior within 10 s (Figure 3). Introducing all of above *TRPA1* transgenes improved the impaired nociceptive behavior of *trpA1*^1^ (either *P* < 0.02 or *P* < 0.03); however, TmTRPA1b and VdTRPA1S appeared to rescue the phenotype to a lesser extent than did DmTRPA1 and VdTRPA1L. More than 20% of the larvae showed response latency of over 10 s. Nevertheless, *trpA1*^1^ expressing VdTRPA1S, but not those expressing TmTRPA1b, showed a statistically significant difference from the wild type (*P* < 0.03; Figure 3). These results suggest that only heat-sensitive mite TRPA1 channels could effectively complement the functions of DmTRPA1 for the thermal nociceptive behavior of fruit fly larvae.

### Critical roles of ARs 6 and 7 for the heat-sensitivity of VdTRPA1L

**Figure 4:**
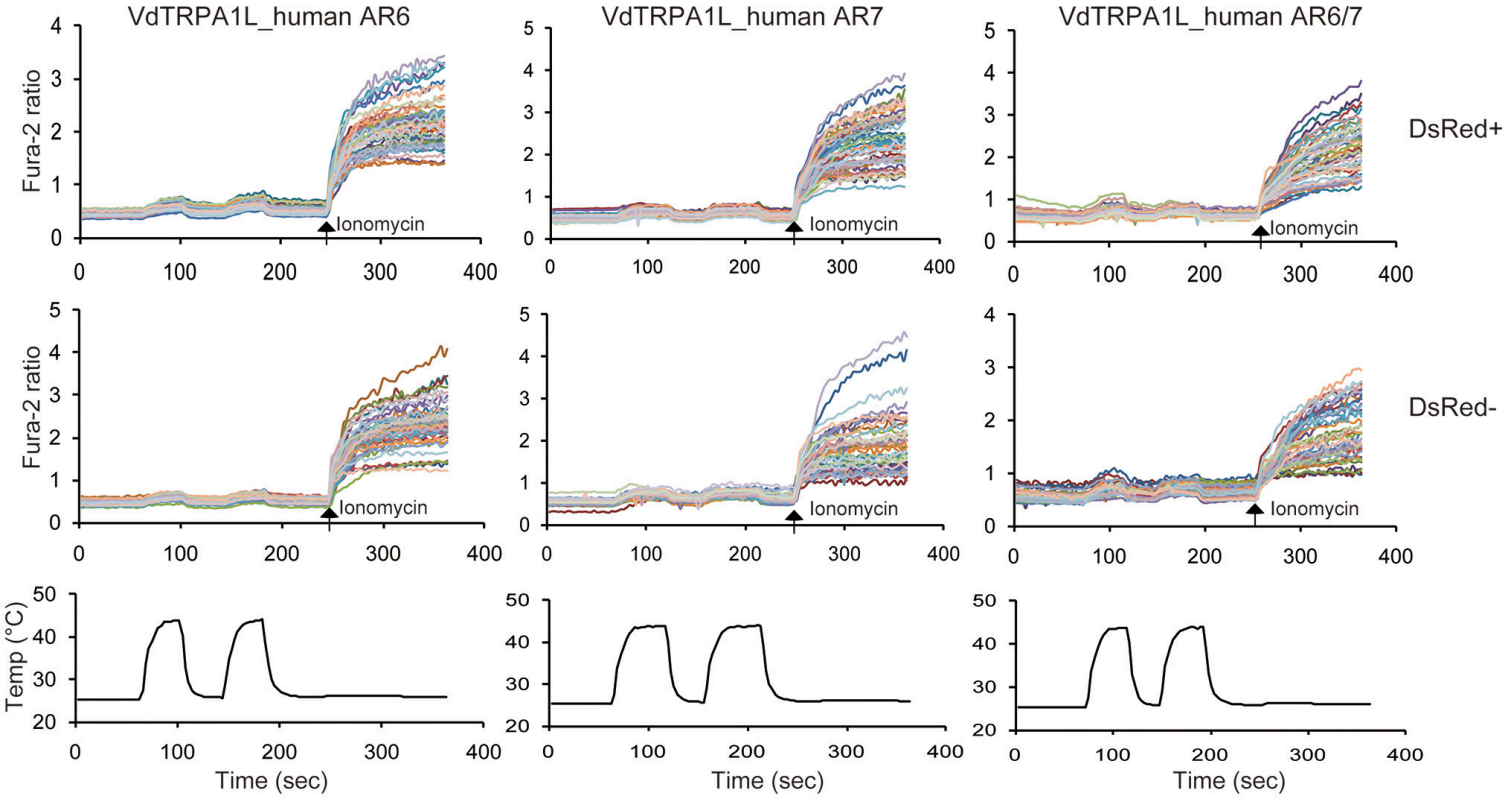
**Loss of heat-activation with the chimeric VdTRPA1L channels**. Two upper traces indicate the changes of intracellular calcium level in transfected (DsRed+) and non-transfected (DsRed-) cells with three chimeric VdTRPA1L channels in which the AR6, AR7, or ARs 6 and 7 were replaced by the corresponding ARs of HsTRPA1 (VdTRPA1L_human AR6, VdTRPA1L_human AR7, and VdTRPA1L_human ARs6/7) as well as DsRed by heat. Each line represents the intracellular calcium level in the individual cell measured by calcium imaging. The arrows show the time points when 5 μM ionomycin was added. The bottom traces show the changes of bath temperature by time (sec, second).

The alignment of six heat-sensitive arthropod TRPA1 channels demonstrates that there are three highly conserved blocks of amino acid sequences (Supplementary Data sheet). One corresponds to the ARs 6 and 7, another to the S4–S5 linker plus the part of S5, and another to part of S6 plus the TRP-like domain. The last two conserved regions are considered to link the structural alteration of S1–S4 sensor domain to opening the lower gate of the channel (Cao et al., 2013; Liao et al., 2013; Paulsen et al., 2015). The overall conservation of S1 to S6 is low (for example, 40% identity and 59% similarity with a 19% gap between DmTRPA1 and VdTRPA1L), except for the selectivity filter and the lower gate of the channels (Supplementary Data sheet). The ARs are more conserved in general (for example, 55% identity and 74% similarity with a 0.9% gap between DmTRPA1 and VdTRPA1L), and the ARs 6 and 7 represent the longest stretch of conserved amino acid sequences among them (Supplementary Data sheet). These suggest that amino acid conservation in ARs 6 and 7 would be critical for the heat-activation of arthropod TRPA1 channels. To test this hypothesis, we examined the heat-sensitivity of chimeric VdTRPA1L in which the AR6, AR7, or both ARs 6 and 7 are swapped with the corresponding ARs of HsTRPA1 which was shown to be heat-insensitive (Cordero-Morales et al., 2011). As shown in Figure 4, all of the chimeric VdTRPA1L channels become heat-insensitive since the subtle increase of Fura-2 ratio by heat was indistinguishable between the transfected (DsRed positive) and non-transfected (DsRed negative) cells. These results suggest that the ARs 6 and 7 of arthropod TRPA1 channels play critical roles for the heat activation.

## Discussion

### Honey bee ectoparasitic mite TRPA1 channels are activated by heat and rescue the behavioral defects of a *D. melanogaster* trpA1 mutant

VdTRPA1L appears to be opened by a temperature increase, but not by a decrease, and does not have a specific threshold temperature for activation (Figures 1D,E). This temperature gating property is quite different from previously characterized thermo-sensitive TRP channels, which exhibit specific threshold temperatures for activation (Voets, 2012). Because *D. melanogaster painless* (Pain) expressed in HEK293 cells exhibited a specific temperature threshold for activation (42.6–44.1°C), as the endogenous Pain in *D. melanogaster* larvae does (Tracey et al., 2003; Sokabe et al., 2008), the lack of a specific threshold temperature for VdTRPA1L activation is unlikely to be an artifact of the heterologous expression in HEK293 cells. Moreover, as we previously reported, *Nasonia vitripennis* (jewel wasp) HsTRPA shares this same lack of threshold as VdTRPA1L (Matsuura et al., 2009). Together with heat-sensitive insect TRPA1 channels (Hamada et al., 2008; Kang et al., 2012; Sato et al., 2014; Wei et al., 2015; Fu et al., 2016) and three TmTRPA1 isoforms, most arthropod TRPA1 channels appear to be activated by heat. Because VdTRPA1L and TmTRPA1b can be functional substitutes for DmTRPA1 for adult thermotactic and larval thermal nociception behaviors (Figures 2, 3), the functions of TRPA1 as a direct thermosensor or downstream component of the signaling pathway activated by thermal stimuli must be evolutionarily well-conserved between arthropods. Nevertheless, VdTRPA1L and TmTRPA1b do not appear to rescue the temperature-related behavioral defects of the *trpA1* mutant as DmTRPA1 does (Figures 2, 3). This could be, for example, due to the fact that VdTRPA1L (most likely TmTRPA1b as well) does not have a specific threshold temperature for heat activation, whereas DmTRPA1 is activated at 27–30°C (Kang et al., 2012). Heat-insensitive VdTRPA1S partially rescued the impaired thermal nociceptive behavior of *trpA1*^1^ mutant larvae (Figure 3), suggesting that it may stimulate the endogenous signaling pathway elicited by Pain when opened by high temperatures (Tracey et al., 2003). Both DmTRPA1 and Pain are expressed in the mdIV nociceptive neurons of larvae and are necessary for responding to high temperatures in larvae and adults (>40°C; Tracey et al., 2003; Neely et al., 2011; Zhong et al., 2012). DmTRPA1 also acts downstream of a phospholipase C–dependent signaling cascade, which is necessary for fruit fly larvae to discriminate between the optimal temperature of 18°C and higher temperatures up to 24°C (Kwon et al., 2008). This temperature range is below the threshold temperature for heat activation of DmTRPA1 (See above). These results suggest that DmTRPA1 can be gated by stimulation of the intracellular signaling pathway independently from heat, and the same may happen with the heat-insensitive VdTRPA1S. This effect would be similar to our previous observation that chemo-insensitive VdTRPA1S stimulated the repression of fruit flies' proboscis extension response elicited by 5 mM carvacrol (Peng et al., 2015a). The detailed channel properties of all arthropod TRPA1 channels have been characterized by heterologous expression systems (either with HEK293 cells or *Xenopus* oocytes), including in the present study, and thus these data need to be carefully interpreted as these channels may not always be the same *in situ*. Thus, the possibility that VdTRPA1S is activated by temperature fluctuations and/or chemical compounds in *Varroa* mites cannot be completely ruled out.

### Physiological functions of the honey bee ectoparasitic mite TRPA1 channels

What are the potential physiological roles of thermosensitivity of TRPA1 channels for *V. destructor* and *T. mercedesae*? Except for distribution by honey bee swarms or foragers, these mites spend their entire lives within the dark hive at a constant temperature of around 30°C by associating with either honey bee adults or larvae. Because honey bees often increase their thorax temperature to nearly 40°C to maintain the hive temperature during winter (Fahrenholz et al., 1989; Stabentheiner et al., 2003), the local temperature inside the hive increases as a result. TRPA1 channels may enable *Varroa* and *Tropilaelaps* mites to sense such temperature increases of the honey bee host and the local environment. Alternatively, the mites may depend on TRPA1 channels to find the brood nest, where the temperature is tightly maintained at 32–36°C to support optimal development of larvae and pupae (Kronenberg and Heller, 1982; Fahrenholz et al., 1989; Tautz et al., 2003). A similar principle may operate when *Varroa* mites switch from the phoretic (associating with adult bees) to the reproductive phase (associating with larvae).

Consistent with the habitat of honey bee mites, the preferred temperature of *Varroa* mites has been shown to be around 32.6°C (Le Conte and Arnold, 1988), which is significantly higher than that of the fruit fly (Figure 2; Sayeed and Benzer, 1996). Likewise, nociceptive high temperatures for the mites may also be different from that of the fruit fly (>40°C; Tracey et al., 2003; Zhong et al., 2012). Thus, these temperature-related behaviors of arthropods are likely to depend not only on TRPA1 and Pain but also on other temperature sensors such as Pyrexia (Lee et al., 2005) and Gr28b (Ni et al., 2013), as well as on the metabolic rate of the animal (Takeuchi et al., 2009).

### Conservation of heat sensitivity among arthropod TRPA1 channels despite their amino acid sequence diversity

Amino acid sequences of DmTRPA1 and *B. mori* TRPA1 (from the AR1 to C-terminal end) share 70% identity and 81% similarity with a 3% gap. The comparison between DmTRPA1 and VdTRPA1L shows only 48% identity and 66% similarity with a 7% gap. Nevertheless, the above three TRPA1 channels are activated by heat (Figure 1; Hamada et al., 2008; Kang et al., 2012; Sato et al., 2014), suggesting that the heat-sensitivity of TRPA1 channels can tolerate various amino acid changes. This may suggest that a relatively small number of specific amino acids is necessary for the heat-sensitivity of these channels. Three domains are particularly well-conserved between arthropod TRPA1 channels (Supplementary Data sheet), and we show one of them, ARs 6 and 7, are critical for the heat-sensitivity of VdTRPA1L (Figure 4). Intriguingly, a previous study also showed that the temperature directionality of mouse TRPA1 activation can be reversed by single amino acid changes in AR6. Furthermore, G276N mutation in AR6 of DmTRPA1 made the channel heat-insensitive (Jabba et al., 2014). These findings suggest that amino acid conservation in ARs 6 and 7 would be critical for the heat-activation of arthropod TRPA1 channels. Nevertheless, they are not sufficient to make the TRPA1 channel heat-sensitive as VdTRPA1S containing the ARs 6 and 7 is not activated by heat (Figure 1), a heat-insensitive mouse TRPA1 in which the AR6 was replaced with DmTRPA1 AR6 is not functional (Jabba et al., 2014), and two DmTRPA1 isoforms with ARs 6 and 7 (dTRPA1-B and dTRPA1-C) are heat-insensitive (Zhong et al., 2012). These results demonstrate that other parts of the channel are also involved in the heat sensitivity of TRPA1. In fact, replacing ARs 10–15 of the heat-insensitive human TRPA1 with those of DmTRPA1 made the chimeric channel heat sensitive (Cordero-Morales et al., 2011). Considering the large evolutionary distance between insects and mites (>410 Mya; Grbic et al., 2011), the heat sensitivity of arthropod TRPA1 channels would be under strong purifying selection. Such signs can be detected, for example, in the conserved amino acid sequence of ARs 6 and 7. It will be important to test more arthropod TRPA1 channels to confirm this hypothesis.

## Author contributions

GP, MK, XD, and TL conducted all experiments. MT and TK designed the experiments. TK wrote the manuscript.

## Funding

This work was supported by 2012 Suzhou Science and Technology Development Planning Programme (Grant#: SYN201213) and Jinji Lake Double Hundred Talents Programme to TK.

### Conflict of interest statement

The authors declare that the research was conducted in the absence of any commercial or financial relationships that could be construed as a potential conflict of interest.
