# Supplementary material for: TRPA1 Channels in Drosophila and Honey Bee Ectoparasitic Mites Share Heat Sensitivity and Temperature-Related Physiological Functions
Source: Front Physiol. 2016 Oct 5;7:447. doi: 10.3389/fphys.2016.00447 (PMC5050203; doi:10.3389/fphys.2016.00447)
Supplement: Supplementary file 1 [file DataSheet1.DOCX]

VdTRPA1L NSPNRILKAAEKGNMAEFQRLLAADERRLQFRDSRGRQAIHHAAAHNRINILEFILNRPN

TmTRPA1 NSPNRILKAAEKGNMAEFQRLLAADERRMQFRDSRGRQAIHHAASHDRINILEFILSRPN

BmTRPA1 -TIEELFQAAESGNVDDFMRLYLSEPSRLAVRDGRGRTAAHQASARNNTNILHFINNY-A

HaTRPA1 ESPFRILRAAESGNVEDFMRLYLSEPSRLAVRDGRGRTAAHQAAAKNNTNILHFINNY-G

DmTRPA1 ------LEAAESGNLDDFKRLFMADNSRIALKDAKGRTAAHQAAARNRVNILRYIRDQ-N

AgTRPA1 DSPYRILRAAEAGNLEEFIRLYEGDNNRLSVKDSKGRTAAHQAAARNRVNILTFIHGQ-G

: *** **: :* ** .: *: ..*..** * *:*::.:. *** :* .

VdTRPA1L VDLRPLDNEGNTPLHSACINGAAQAIAFFINNDPEQLQCFNKEHQSPLHVATQLNKVASL

TmTRPA1 AELRPVDNEGNTPLHSACTTGAAKAIAFFIQHDPEQLECYNKENQSPLHLATQLNKVASL

BmTRPA1 GDLNAKDNAGNTPLHVAVENEALDAIEYLLQQ-HVETSVLNEKCQAPIHMATELNKVSVL

HaTRPA1 GDLNAKDNFGNTPLHVAVENVALDAIEFLLQH-RVDTSILNDKSQGALHLATELNRVSVL

DmTRPA1 GDFNAKDNAGNTPLHIAVESDAYDALDYLLSI-PVDTGVLNEKKQAPVHLATELNKVKSL

AgTRPA1 GNLNAQDMVGNTPLHTAVENDSLDALEFLLKI-PVATNVLNEKKLAPVHLATEQNKVHAL

::.. * ****** * . : .*: :::. *.: ..:*:**: *.* *

VdTRPA1L KALCNFKDKIDVEQRSKHGRTALHIACISDNAEAALVLLRDFAASPKTTCDNGFYPIHEA

TmTRPA1 TALCDFKSQIDVEQKSKHGRTALHLACISDHADAALILLRDFGASPKTTCDNGFYPIHEA

BmTRPA1 QVFAKYKSLFNVNQGGEHGRTALHFAAIHDHDLCAKILITDLDAEWKKPCNNGYYPIHEA

HaTRPA1 QTFTKYKDQFNVELGGEHGRTALHFAAIHDHDICAKILITDLGAECKRACNNGYYPIHEA

DmTRPA1 RVMGQYRNVIDIQQGGEHGRTALHLAAIYDHEECARILITEFDACPRKPCNNGYYPIHEA

AgTRPA1 QVMGKYREVIDIQQGGEHGRTALHLAAIYDNEECARILISEFGACPRKPCNNGYYPIHEA

.: .:.. :::: .:*******:*.* *: .* :*: :: * . .*:**:******

**ARs 6 and 7**

VdTRPA1L AKNASANAMRVLLEFCESIGIPRADMMKLFDADGNVPLHSAVHAGDLKAVELCLESGALI

TmTRPA1 AKNASANAMRALLEFCESIGIPRAEMMKLFDADGNVPLHSAVHAGDLKAVELCLESGALI

BmTRPA1 AKNASSRTIEVFLQWGEQRGCTREQMISLYDNEGNVPLHSAVHGGDIKAVELCLRSGAKI

HaTRPA1 AKNASSRTMEVFLQWGESKGCTRESMISLYDNEGNVPLHSAVHGGDIKAVELCLKSGAKI

DmTRPA1 AKNASSKTMEVFFQWGEQRGCTREEMISFYDSEGNVPLHSAVHGGDIKAVELCLKSGAKI

AgTRPA1 AKNASSKTMEVFFQWGESKGCTREEMISFYDSEGNVPLHSAVHGGDIKAVELCLKSGAKI

*****:.:: .:::: *. * .* .*:.::* :**********.**:******* *** *

VdTRPA1L STQQHDLSTPVHLACSQGAIDIVKLMFRCQPDQKMGCLTCADAQNMTPLHCAAMFDHVEL

TmTRPA1 STQQHDLSTPVHLACSQGAMDIVKLMFRCQPDQKMGCLTCSDAQNMIPLHCAAMFDHVDL

BmTRPA1 STQQHDLSTPVHLACAQGALDIVKLMFTMQPKEKHACLTSCDVQKMTPIHCAAMFDLPEI

HaTRPA1 STQQYDLSTPVHLACAQGALEIVKLMFTMQPTEKLACLTSCDVQKMTPVHCAAMFDHPDI

DmTRPA1 STQQHDLSTPVHLACAQGAIDIVKLMFEMQPMEKRLCLSCTDVQKMTPLHCASMFDHPDI

AgTRPA1 STQQHDLSTPVHLAAAQGAIEIVKLMFRMQPLEKRISLNCTDIQKMTPLHCAAMFDHPEI

****:*********.:***::****** ** :* .*.. * *:* *:***:*** ::

VdTRPA1L VNFLVDEGASLNATDKEGRSVVLLAAARSAWKTVTSVLKLGADLGLQRDNQGRNLLHHIV

TmTRPA1 VNFLVDEGASLNATDKEGRSVLLLAAARSAWKTVASVLKLGADLGLQRDNQRRNLLHHIV

BmTRPA1 VNYLINEGSDINPLDKERRSPLLLAASRAGWRTVHTLIRLGADIQL-KDINSRNVLHLVV

HaTRPA1 VNYLISEGSDINPLDKERRSPLLLAASRAGWRTVHTLIRLGADIEL-KDINSRNVLHLVV

DmTRPA1 VSYLVAEGADINALDKEHRSPLLLAASRSGWKTVHLLIRLGACISV-KDAAARNVLHFVI

AgTRPA1 VEYLVKEGADINAMDKEKRSPLLLSSSRGGWRTVMALIRLGANISL-KDANSRNVLHLVI

*.:*: **:.:*. *** ** :**:::*..*.** ::.*** : : .* **:** ::

VdTRPA1L LSSGSIEEFTTSI------NDRLEDFIQLLNERDSYGCTALHYAARNGQLKSIQSLIALG

TmTRPA1 LSGGSIEEFTLSL------NDRLDEFIQLLNERDTYGCTALHYAARNGQLKSIQSLITLG

BmTRPA1 MNGGRLEDFAAS-CK----DHCEKSLLQLLNEKDNTGCSPLHYASREGHIRSLENLIRLG

HaTRPA1 MNGGRLEDFAAT-CKLTLQNRCDKSLAQLLNEKDSAGCSPLHYASREGHIRSLENLIRLG

DmTRPA1 MNGGRLTDFAEQVAN----CQTQAQLKLLLNEKDSMGCSPLHYASRDGHIRSLENLIRLG

AgTRPA1 MNGGCLDEFAKEVCR----TQSEIYLLQLLNEKDDAGCSPLHYASREGHIRSLENLIRLG

:..* : :*: : ****.* **:.****:*:*::.*::.** **

VdTRPA1L ASVNLKNNENQSPLHFAAMFGRFNTVRHLLDSKKGHLIINEMDGMGKTPLHLASQCGHVR

TmTRPA1 ASVNLKNNDNQSPLHFAAMFGRLNTVRHLLDSKKGHLIINEMDGQGKTPLHIASQCGHVR

BmTRPA1 ACINLKNNNNESPLHFAARYGRFHTACQLLDSDKGTFIINESDGEGLTPLHIASREGHTR

HaTRPA1 ACINLKNSNNESPLHFAARYGRYHTACQLLDSDKGTFIINESDGEGLTPLHIASREGHTR

DmTRPA1 ACINLKNNNNESPLHFAARYGRYNTVRQLLDSEKGSFIINESDGAGMTPLHISSQQGHTR

AgTRPA1 ACINLKNNNNESPLHFAARYGRYNTVRQLLDSEKGTFIINESDGEGLTPLHIASQQGHTR

*.:****.:*:******* :** :*. :****.** :**** ** * ****::*. **.*

VdTRPA1L VVHLLLVKGALLHRDHKGRTPLHYASMNGYNNTMDQLLAVHSHLLDQTDRDGNTALHMAA

TmTRPA1 VVHLLLVKGALLHRDHKGRTPLHYAAMNGYNNTMDQLLAVHSHLLDQTDRDGNTALHMAA

BmTRPA1 VVHLLLNRGALLHRDHNGRNPLHLAAMSGYTKTIELLHSVHSHLLDQIDKDGNTSLHLAT

HaTRPA1 VVHLLLNRGALLHRDHNGRNPLHLAAMSGYTQTIELLHSVHSHLLDQSDKDGNTPLHLAT

DmTRPA1 VVQLLLNRGALLHRDHTGRNPLQLAAMSGYTETIELLHSVHSHLLDQVDKDGNTALHLAT

AgTRPA1 VVQLLLNRGALLHRDHNGRNPLHLAAMSGYRQTIELLHSVHSHLLDQVDKDGNTALHLAT

**:*** .********.**.**: *:*.** :*:: * :******** *.****.**:*:

**End of ARs**

VdTRPA1L MKNRSSTAINLLNLNCKIIKNGLDMTPMDYALHYKHSEVAMAMVIHPSRSDEVMTCQVKT

TmTRPA1 MKNRSSTAVNLLNLSCKITKNGLDMTPMDYALHCKHSEVAMAMVTHPIRSDEIMTCQVKA

BmTRPA1 MENKPSSIALLLSMGCRLSYNNMDMSAIDYAIYYKFPEAALAMVTHEQRAKEVMALRSDR

HaTRPA1 MENKPNSIALLLSMGCRLSYNNLDMSAIDYAIYYKFPEAALAMVTHEHRAKEVMALRSDR

DmTRPA1 MENKPHAISVLMSMGCKLVYNVLDMSAIDYAIYYKYPEAALAMVTHEERANEVMALRSDK

AgTRPA1 MENKPNAVILLLSLGCKLLHNYMDMSAIDYAIYYKYPEAALAMATHEERSSEVMALKSDK

*:*.. : *:.:.*.: * :**:.:***:: *..*.*:**. * *:.*:*: . .

VdTRPA1L YSCLVEGLIAVMPEVMTTVLDRGISKSKMSHDSDEYFVKYSFTCLQ--------NSEGAD

TmTRPA1 YGCLVEGLTAVMPEVMMTVLDRGISKSKMSHDSEEYFVKYSFTSLQ--------NSETAD

BmTRPA1 HPCVTLALIAYMPRVFEAVQDKCITKANCKKDSKSFYIKYSFEAL-CPQL---MDEDGTR

HaTRPA1 HPCVTLALIAYMPRVFEAVQDKCITKANCKKDSKSFYIKYSFKFYQRSKLEVD-ALRLAL

DmTRPA1 HPCVTLALIASMPKVFEAVQDKCITKANCKKDSKSFYIKYSFAFLQCPFMFAKIDEKTGE

AgTRPA1 HPCVTLALIASMPRVFEAVQDNCITKANCKKDSKSFYIRYSFSCLQCPALYAQMDARTGE

: *:. .* * ** *: :* *. *:*:: .:**..:::.***

**Start of S1**

VdTRPA1L VVPMTD-EPLPVMNIMVRYGREELLSHPLSVKYLETKWNAYGIYFHILNLMVYTVFLCFL

TmTRPA1 VVPMTD-EPLPVMNIMVRYGREELLSHPLSVKYLETKWNAYGMYFHILNLMVYTVFLGFL

BmTRPA1 KSQQAQQIPLPALNAMVAHGRVELLAHPLSQKYLQMKWNSYGKYFHLVNVLFYCIFLIFV

HaTRPA1 NDPKYRPEPLCVINAMVAHGRVELLAHPLSQKYLQMKWHSYGKYFHLANLLFYCIFLIFV

DmTRPA1 SITTASPIPLPALNTMVTHGRVELLAHPLSQKYLQMKWNSYGKYFHLANLLIYSIFLVFV

AgTRPA1 AVQISKPIPLPALNAMVAHGRVELLAHPLSQKYLQMKWNSYGKYFHLANLLFYSVFLFFV

** .:* ** :** ***:**** ***: **::** ***: *::.* :** *:

VdTRPA1L TLNAVQLMQD-----NKKRHQ---------------------------GISSFEVADAFV

TmTRPA1 TLNAVQLMQD-----NKKRHH---------------------------GVSGLEVADAFA

BmTRPA1 TVYSYLLMEHVNPINKRERSR---------------------------VGDVYYNYTATN

HaTRPA1 TVYTYLLMMNADASSPSKKSA-----KLCT------------------NLSDSANVDREN

DmTRPA1 TIYSSLMMNNIELKAGDNKTM----SQYCNMGWEQLTM----------NLSQNPSVASQI

AgTRPA1 TLFTSQLMRNATPIGHTDGNHTQAAGTPVDSGQHILALRSTIARSKGYNLGTVANVSSSV

*: : :* . .

VdTRPA1L RPSL----------ATLSSMTSVVVLSFVLVNVVKEAFQLIQQRTKYFVDVMNILEWTLY

TmTRPA1 RPSI----------ATLSSMTSVIVLSFVLFNVVKEAFQLIQQRTKYFVDVMNILEWTLY

BmTRPA1 RTQTNWDIDADFEAILIMYTSSVVIMVYISVCMMREAYNLKQQKWHYIVDPSNLVSWTLY

HaTRPA1 KSDKNIVFNPDFESKVAMYTSTVAILVYNSICLVREAYNVKQQKWHYMVDPSNLVSWLLY

DmTRPA1 RLDS---CEERINRTTAILFCAVVIVVYILLNSMRELIQIYQQKLHYILETVNLISWVLY

AgTRPA1 APPT---IEEQMEVTTTTLVSGIGIIIYIVVNALRELVQVYQQKWHYLLEPNNFISWILY

: :: : . :.* :: **. :*::: *::.* **

**S4-S5 linker and the part of S5**

VdTRPA1L LAAGLMALSQL-TRIEEKSYQNIVAAVAVFLAWFNYLLFLQRFNRVGLYVVMFLEILSTL

TmTRPA1 LASGLMALSQL-TRTEEKSYQNIVAALAVFLAWFNYLLFLQRFNRVGLYVVMFLEILSTL

BmTRPA1 ISATITVFPTL--YGHYSNYQFSAASITVFLSWFELLLLLQRFDQVGIYVVMFLEILQTL

HaTRPA1 ISSTLMVFPTI--FGYFDEIQFSAASITVFLSWFELLLLLQRFDQVGIYVVMFLEILQTL

DmTRPA1 ISALVMVTPAFQPDGGINTIHYSAASIAVFLSWFRLLLFLQRFDQVGIYVVMFLEILQTL

AgTRPA1 TSALIMIWPMF-SSGMCFSINYSAASITVFLSWFNLLLFLQRFDQIGIYVVMFLEILQTL

:: : . : : .*:::***:** **:****:.:*:*********.**

VdTRPA1L LRVVMVFSVLIIAFGLSFHILLARVDLTVQQPIFDHRTGNVTFKPISLGDKGFHTPLVSL

TmTRPA1 LRVVMVFSVLIIAFGLSFHILLARVELSLKQPVLDARTGNVTFKAISLSDKGFHSPLVSL

BmTRPA1 IKVLMVFSILIIAFGLAFYILLSK-------------GNHLSFSNIPI----------AL

HaTRPA1 IKVLMVFSILIIAFGLAFYILLSK-------------GHHLSFNSIPM----------SL

DmTRPA1 IKVLMVFSILIIAFGLAFYILLSK--------IIDPQPNHLSFSNIPM----------SL

AgTRPA1 IKVLIVFSILIIAFGLAFYILLSK--------VSEPQVNHLSFSSIPM----------SL

:.*::***:*******:*:***:. :::*. *.: :*

VdTRPA1L IRVGTMMLGELDFLGTYLRPLRNLENSTWYQIVAATLFLVA-FIILMPILLMNLLIGLAV

TmTRPA1 IRVGTMMLGEIDFLGTYLRPLRNLENSTWYQIVTATLFLVA-FIILMPILLMNLLIGLAV

BmTRPA1 MRTFAMMLGELDFVGTYVQPYYKDDSDIILPFPIPTFIILALFMILMPILLMNLLIGLAV

HaTRPA1 MRTFAMMLGELDFVGTYVQPYYKSETDILLPFPMPTFFILGIFMVLMPILLMNLLIGLAV

DmTRPA1 LRTFSMMLGELDFVGTYVNTYYRDQ----LKVPMTSFLILSVFMILMPILLMNLLIGLAV

AgTRPA1 VRTFSMMLGEMDFVGTYVQPYHVGD----LPFPFPSFVILCLFMILMPILLMNLLIGLAV

:*. :*****:**:***:.. : . .::.:: *::***************

**The part of S6 and TRP-like domain**

VdTRPA1L GDIETVRRNAQLKRLTMQVRLHTDLERKLPKRILDSVDRTEVSIHPNQKGTGNIFWRTI-

TmTRPA1 GDIETVRRNAQLKRLTMQVRLHTDLERKLPKKILEAVDRTEMCIYPNQKGTGNIMWRTI-

BmTRPA1 GDIESVRRNAQLKRLAMQVVLHTELERKLPAFLLEKVDKLELIEYPNNKKCKLGFLDLIL

HaTRPA1 GDIESVRRNAQLKRLAMQVVLHTELERKLPAILLEKVDKDELIEYPNNKKCKLGFLDLIL

DmTRPA1 GDIESVRRNAQLKRLAMQVVLHTELERKLPHVWLQRVDKMELIEYPNETKCKLGFCDFIL

AgTRPA1 GDIESVRRNAQLKRLAMQVVLHTELERKLPQMWLEMVDKMELIEYPNEKKCKLGFLDSVL

****:**********:*** ***:****** *: **. *: :**:. : :

VdTRPA1L KRWFGSPRNNG--NGVQSRNSKNLHKSFTQFIESNINDIHQQLGRQKDKIREISRNMEMQ

TmTRPA1 KRWFGSPRNNVTSGGGGGANGKNLSKSFTQQIESNINEIQEQLSRQKDKIREISKNLELQ

BmTRPA1 HKWFCNPFTED--TGLD-----------LV-LENNDDYVTEQMEKQKRKFREMQNVLDQQ

HaTRPA1 RKWFCNPFTDD--TGLD-----------LV-LESSEDYITAELEKQKRKLRDISLVLEQQ

DmTRPA1 RKWFSNPFTED--SSMD-----------VISFDNNDDYINAELERQRRKLRDISRMLEQQ

AgTRPA1 RKWFCNPFTDDYKGGID-----------YV-LENTEDYVAVELEKQKRKLRDIGTALDAQ

..** .* .: . ::.. : : :: .*. *:*:: :: *

VdTRPA1L VTLLRLLVSKMEIKSEAEELDEGE-------------------FNSASLARDTAE--QVD

TmTRPA1 LTLVRLLVSKMEIKSEAEELDEGE-------------------FNSANLARDTTE--QLD

BmTRPA1 YLLLRLIVQKMEIKTEADDVDEGVSPNDTKVV---------PRWSSHRNRKKLHSARAAS

HaTRPA1 HTLVRLIVQKMEIKTEADDVDEGVSPGDTRVI---------PRWSSPRIRKKLRT--ASS

DmTRPA1 HHLVRLIVQKMEIKTEADDVDEGISPNELRSVVG-LRSAGGNRWNSPRVRNKLRA--ALS

AgTRPA1 HQLLRLIVQKMEIKTEADDVDEGVSTSDLKASSGLLTGTRSSRWSSPRIRKKLGA--TLS

*:**:*.*****:**:::*** :.* .. .

VdTRPA1L GDSATDIK

TmTRPA1 GQTDVK--

BmTRPA1 FNKST---

HaTRPA1 FNKGG---

DmTRPA1 FNKSM---

AgTRPA1 FNKSIGK-

:.

**Supplementary Figure 1 Amino acid sequence alignment of six heat-sensitive arthropod TRPA1 channels**

Amino acid sequences of *Varroa destructor* TRPA1L (VdTRPA1L), *Tropilaelaps mercedesae* TRPA1 (TmTRPA1), *Bombyx mori* TRPA1 (BmTRPA1), *Helicoverpa armigera* TRPA1 (HaTRPA1), *Drosophila melanogaster* TRPA1 (DmTRPA1), and *Anopheles gambiae* (AgTRPA1) are aligned by MUSCLE. Three blocks of highly conserved amino acid sequences are highlighted with yellow (ARs 6 and 7), green (S4-S5 linker and the part of S5), and light blue (the part of S6 and TRP-like domain). End of ARs and start of S1 are also marked with purple and gray, respectively. Identical amino acids between the six channels are indicated by asterisks and the similar amino acids are shown by either (.) or (:).
